# Adaptation of the spiders to the environment: the case of some Chilean species

**DOI:** 10.3389/fphys.2015.00220

**Published:** 2015-08-11

**Authors:** Mauricio Canals, Claudio Veloso, Rigoberto Solís

**Affiliations:** ^1^Departamento de Medicina and Programa de Salud Ambiental, Escuela de Salud Pública, Facultad de Medicina, Universidad de ChileSantiago, Chile; ^2^Departamento de Ciencias Ecológicas, Facultad de Ciencias, Universidad de ChileSantiago, Chile; ^3^Departamento de Ciencias Biológicas Animales, Facultad de Ciencias Veterinarias y Pecuarias, Universidad de ChileSantiago, Chile

**Keywords:** spiders, ecophysiology, energetics, mediterranean region, lung

## Abstract

Spiders are small arthropods that have colonized terrestrial environments. These impose three main problems: (i) terrestrial habitats have large fluctuations in temperature and humidity; (ii) the internal concentration of water is higher than the external environment in spiders, which exposes them continually to water loss; and (iii) their small body size determines a large surface/volume ratio, affecting energy exchange and influencing the life strategy. In this review we focus on body design, energetic, thermal selection, and water balance characteristics of some spider species present in Chile and correlate our results with ecological and behavioral information. Preferred temperatures and critical temperatures of Chilean spiders vary among species and individuals and may be adjusted by phenotypic plasticity. For example in the mygalomorph high-altitude spider *Paraphysa parvula* the preferred temperature is similar to that of the lowland spider *Grammostola rosea*; but while *P. parvula* shows phenotypic plasticity, *G. rosea* does not. The araneomorph spiders *Loxosceles laeta* and *Scytodes globula* have greater daily variations in preferred temperatures at twilight and during the night, which are set to the nocturnal activity rhythms of these species. They also present acclimation of the minimum critical temperatures. *Dysdera crocata* has a low preferred temperature adjusted to its favorite prey, the woodlouse. Spider metabolic rate is low compared to other arthropods, which may be associated with its sit and wait predatory strategy particularly in primitive hunter and weavers. In mygalomorph spiders the respiratory system is highly optimized with high oxygen conductance, for example *G. rosea* needs only a difference of 0.12–0.16 kPa in the oxygen partial pressure across the air-hemolymph barrier to satisfy its resting oxygen consumption demands. Water loss is a significant stress for spiders. *Paraphysa parvula* shows an evaporative water loss 10 times more than usual when the temperature approaches 40°C and the participation of book lungs in this loss is about 60%. This species and others show seasonal changes in water loss accounted for by changes in cuticle permeability. The case of Chilean spiders shows how the ecophysiology in spiders is associated to their design and body size and how is affected by fluctuating Mediterranean environments, suggesting that the adaptive process can be seen as a route of optimizing the use of energy to cope with environmental restrictions imposed by the interaction with the terrestrial environment and lifestyle.

## Introduction

The main objective in ecophysiology is the analysis of the physiological mechanisms of ecological and evolutionary significance, determining how physiology affects and is affected by the distribution and abundance of animals in space and time, and the study of patterns and processes by which physiological variability originates and/or persists. Another objective is to determine how some animal species can exploit extreme environments such as high altitude and extreme deserts, and how physiological constraints are reflected in differential biological adaptations (Bozinovic and Canals, 2008).

The balance between acquisition and energy expenditure depends on the interaction between intake of energy/matter, digestive processing, allocation, and metabolic rate. Structural features, behavioral, physiological, and biochemical components, foraging, and digestion and the thermodynamic efficiency of use and production patterns of energy/matter are under selective pressure. For example, in mammals survival in low temperatures depends on the thermogenic ability of individuals, while the number and quality of a litter depends on the rate of energy production in milk exported by the female (Veloso and Bozinovic, 1993, 2000a,b; Veloso, 2003). So natural selection would favor those life strategies to ensure that energy costs are balanced with the energy inputs assimilated from food (Bozinovic et al., 1985).

One might then expect that the organisms that maximize the difference between energy input and operating costs would be favored by selection, since energy losses would be detrimental to the survival and reproduction functions. Seen this way, one could understand the adaptive process as the route of optimization in the use of energy subject to the restrictions imposed by the environment, whether given by the way of life, a restriction of space or other factors (Bozinovic and Canals, 2008).

In this review we analyze the thermal and water aspects of spider physiology of Chilean spiders from fluctuating Mediterranean environments and how they are associated and conditioned by their own design.

## Design and body size

Arthropods have colonized terrestrial environments in the Late Cambrian-Early Ordovician, 500 million years ago, although previous evidence suggesting an origin in the Middle Ordovician and bodies of terrestrial arthropods are from the Silurian, about 400 million years ago. In this long evolutionary time this group has had to adapt to the challenges posed by the transition from water to land (MacNaughton et al., 2002)

Spiders are ectothermal species, i.e., the internal temperature depends on heat transferred from the external environment; and they are also poikilothermic, i.e., body temperature varies, but can be regulated behaviorally. All metabolic activities depend on temperature, which is expressed through the relationship of Van't Hoff-Arrhenius, valid for all living organisms: $M=c\cdot m_{b}^{3∕4}\cdot e^{-E_{i}∕kT}$, where c is an arbitrary constant, *m*_*b*_ is the body mass, *Ei* is the average energy of activation of biochemical enzymatic reactions of metabolism, *k* is the Boltzmann constant and *T* the temperature in degrees Kelvin (Gillooly et al., 2001). This relationship shows that there is an extreme dependence of metabolic rate on temperature in poikilothermic animals, increasing exponentially with temperature.

To colonize terrestrial environments these animals must overcome three fundamental issues: (i) terrestrial habitats have large fluctuations in temperature and humidity, in the availability of drinking water, in wind speed and many other relevant parameters for water and heat balance; (ii) the concentration of water molecules in the internal medium is higher than in the external environment, which makes them continually exposed to water loss; and (iii) body size is generally small, from millimeters to centimeters and from milligrams to grams, which results in a high surface/volume ratio that makes them extremely susceptible to environmental changes of temperature and humidity (Nentwig, 2013).

Arthropod body size is generally small and presents a wide range of size variation. For example, the ratio between the size of a small arthropod (fraction of a milligram) and a 9 kg decapod is 1: 100,000,000, which is one order of magnitude greater than the difference between an elephant and a shrew. In addition to their very small individual sizes, the number of species and individuals in each species is very high, contributing to the accentuated negative slope of the curve that relates body size and number of species. One limitation to large body size in this group is given by the biomechanical limits of strength. While strength and muscle force are proportional to the square of the diameter of the limbs, body mass follows the cube of the linear dimension, which imposes a limit on the maximum size (McMahon, 1983). Thus, body sizes in arthropods have only reached sizes of about 3 m in the Gigantostracos of the Silurian period. In addition, gigantism in arthropods has only been achieved in periods of high environmental oxygen concentration. Thus, giant insect forms developed in the hyperoxic environments of Carboniferous-Permian and in the period from the middle Jurassic-Cretaceous to Tertiary (Dudley, 1998; Falkowski et al., 2005).

The surface of animals is variable in its general appearance; it is modifiable by conduct and participates in such diverse functions as food intake, locomotion, respiratory exchange, protection, water regulation, and heat exchange. These facts explain its high variability and indicate that its morphology is determined by multiple factors. For example, in endothermic animals whose body temperature is maintained above the environmental temperature, the outer surface must minimize heat loss to the environment. Hence animal forms tend to adopt Euclidean, cylindrical or sphere-like geometries. As an example, the Meeh constant (K = A/V^2∕3^, A the area and V the volume of the body) is 10 in mammals, 6 in a cube and 4.84 in a sphere. In contrast, fractal shapes maximize exchange with the environment as this relationship and the relationship between area and perimeter tend to infinity (maximal surfaces). Animals can generally modify their own geometry behaviorally, such as the tendency to the spherical shape of birds and mammals when subjected to cold and “huddling” behavior (Canals et al., 1989, 1997a, 1998, 2011, 2015; Canals, 1998). However, in terrestrial arthropods such as spiders the outer surface cannot be changed because of the existence of the exoskeleton, having to modify it changing their position when necessary.

Dimensional analysis shows that the surface of a body is proportional to the square of the linear dimension (AαL^2^), while the volume is proportional to the cube of the linear dimension (VαL^3^), thus for area AαV^2∕3^; so considering that the density of organisms is about 1, area per unit of body mass (A_mb_) follows A_mb_ α m${}_{\text{b}}^{-1∕3}$, i.e., the smaller the animal the larger the mass-specific surface.

The metabolism of spiders is determined by the shape of the body. Simply put, you could say that since metabolism is generally a surface phenomenon it would follow a curve similar to that of A_mb_, where smaller animals have higher mass-specific metabolism. Dimensional analysis takes us in the same direction; metabolism (MR) is energy (E) per unit time (t), MR = E/t = maL/t α L^3^(L/t^2^)L/t = L^5^/t^3^, where m is the mass, a the acceleration and L the linear dimension. Thus, MR would be proportional to the ratio L^5^/t^3^. According to the theory of biological similarities (Lambert and Teissier, 1927; Günther and Morgado, 1996, 2003; Grossi and Canals, 2015) in mechanical physical phenomena time is proportional to m^1∕3^, i.e., time is proportional to L (t α L), thus metabolism would be MR α L^5^/L^3^ = L^2^ α V^2∕3^ α m${}_{\text{b}}^{2∕3}$ and metabolism per unit mass would be, MR_mb_ = MR/m_b_ α m${}_{\text{b}}^{-1∕3}$, reaching the same result (Grossi and Canals, 2015). However, the exponent 2/3 has undergone extensive discussion and differs from the ¾ exponent that is the most currently accepted, as seen in the Gillooly relationship mentioned above (Gillooly et al., 2001). This is easily seen if we consider that the biological similarities law applies only to certain macroscopic physical mechanical phenomena, while metabolism has to do with microscopic phenomena occurring on surfaces or folded borders or thin vascular networks or arborizations with tubular fractal geometry where the timescale is related to cyclical phenomena of turnover rates. In this case the time scale with m^1∕4^ has been proposed, i.e., to L^3∕4^ (Günther and Morgado, 2003), which leads to a relationship between metabolism and mass with a larger exponent (0.92). Other authors reached the same result considering organisms as area-volume hybrids with fractal transport systems (Sernetz et al., 1985), or considering that life is sustained by the transport of materials through branches that distribute energy and materials (West et al., 1997) under the assumptions that: (i) branch piping systems are fractal-like, (ii) the size of the terminal is an invariant, and (iii) the costs of distributing energy resources is minimal (minimum entropy production), reached the same exponent expected and used by Kleiber and by Gillooly for this relationship (but see Chaui-Berlinck, 2006 for a criticism).

Recently, using physical and allometric principles such as those described above, Grossi and Canals (2015) analyzed the role of energy in sexual size dimorphism of spiders. They proposed that the cost of transport or equivalently energy expenditure and speed are traits under selection pressure in male spiders, favoring those of smaller size to reduce travel costs. The morphology of spiders responds to these selective forces depending upon the lifestyle of the spiders. Climbing and bridging spiders must overcome the force of gravity. If bridging allows faster dispersal, small males would have a selective advantage by enjoying more mating opportunities. In wandering spiders with low population density and as a consequence few male–male interactions, high speed and low energy expenditure or cost of transport should be favored by natural selection. Also, pendulum mechanics showed the advantages of long legs in spiders and their relationship with high speed, even in climbing and bridging spiders. Thus, they proposed that small size compensated by long legs should be the expected morphology for a fast and mobile male spider.

Small body size has other benefits, such as an increase in the surface of oxygen exchange with the environment and an increase in surface area per volume unit which allows greater wind resistance, allowing dispersion and eventually passive flight, but also it brings problems due to easy cooling and water loss and the mentioned implications in metabolic rates.

## Energetics

In arthropods there is variability in the allometric exponent (b) between mass and metabolism (Table 1). In terrestrial arthropods Lighton and Fielden (1995), Lighton et al. (2001) found an exponent *b* = 0.856 for terrestrial arthropods in general and the same exponent for ticks and scorpions, but the intercept for the latter groups is smaller, so that for the same mass the metabolism of ticks and scorpions may be up to 24% less. Previously, Greenstone and Bennett (1980) found in spiders (Araneae) an exponent *b* = 0.710, which is significantly lower than those estimated for other poikilothermic animals. Thus, for example, in a 100 mg spider metabolism would be only 74% of that predicted for other poikilothermic animals. This finding is consistent with previous results of other authors (see Anderson, 1970).

**Table 1:** **Allometric exponent (b) between metabolic rate (MR) and body mass (m_b_): M α m${}_{\text{b}}^{\text{b}}$ in Arthropods (modified from Glazier, 2005)**.

**Taxa**	**Exponent**
**CHELICERATA**	
Arachnida	0.854 ± 0.076
**CRUSTACEA: BRANCHIOPODA**	
Anostraca	0.641 ± 0.306
Conchostraca	0.840
Notostraca	0.803
Cladocera	0.863 ± 0.086
**CRUSTACEA: MAXILOPODA**	
Ostracoda	0.746
Copepoda	0.845 ± 0.060
Cirripedia	0.694 ± 0.114
**CRUSTACEA: MALACOSTRACA**	
**Eucarida**	
Euphausiacea	0.859 ± 0.167
Decapoda	0.718 ± 0.056
**Peracarida**	
Mysida	0.686 ± 0.088
Isopoda	0.723 ± 0.027
Anphipoda	0.719 ± 0.085
Myriapoda	0.756 ± 1.187
Insecta	0.830 ± 0.036

Low resting metabolic rates may be associated with adaptation to environments of low predictability and low prey availability (Anderson, 1970; Greenstone and Bennett, 1980; Prestwich, 1983a,b; Wilder, 2011). Physiologically this could be due to the fact that spiders use hydrostatic pressure for the extension of their appendages, maintaining a posture with constant hydrostatic pressure with a small number of active muscles instead of the permanent use of all muscles with consequent metabolic activity (Carrel and Heathcote, 1976; Anderson and Prestwich, 1982). Low resting metabolic rate may be a factor that allows them to extend their survival without food (Tanaka and Ito, 1982; Canals et al., 2007; Nentwig, 2013). In addition, spiders may reduce their metabolic rate significantly when they experience periods of food limitation (Ito, 1964; Miyashita, 1969; Anderson, 1974; Tanaka and Ito, 1982; Canals et al., 2007; Phillip and Shillington, 2010; Stoltz et al., 2010; Canals et al., 2011). Other aspect of their low energy expenditure strategy is the low and short increase of postprandial metabolism (specific dynamic action) attributed to their external digestion (Nespolo et al., 2011).

As examples of the above, in mygalomorph spiders metabolic rates of 0.018 mlO_2_/gh in *Aphonopelma eutylenum* (Greenstone and Bennett, 1980) and 0.013 mlO_2_/gh in *A. californicum* (Paul et al., 1987) have been reported. In *Grammostola rosea*, a large spider of more than14 g, a metabolic rate of 0.027 ± 0.01 mlO_2_/gh was found, which corresponds to 63.3% of the expected value for other spiders of its body mass. *Paraphysa parvula*, a small mygalomorph of Chile, at 25°C had a metabolic rate of 16.4 ± 7.3% of the expected value for other arthropods of the same body mass (Figueroa et al., 2010). Also, a metabolic depression at 30°C with food deprivation has been reported in *G. rosea* (Canals et al., 2008). The mygalomorphae spider *Euathlus truculentus* increases its metabolism to 85% of that expected value for invertebrates with a peak at 45 min, suggesting that this spider spent most of the energy for digestion in a short period after prey capture, which could be a consequence of external digestion (Nespolo et al., 2011). However, Lighton et al. (2001) proposed that spiders have metabolic rates similar to those of other land arthropods. They proposed that resting metabolic rate may be considered very conservative and that a general allometric rule between body mass and resting metabolic rate may be assessed for all land arthropods except tarantulas (Shillington, 2002, 2005), scorpions and ticks (Lighton et al., 2001).

Some studies have failed to show metabolic differences other than those due to body mass, which could be a consequence of ecological differences between different groups of spiders (Greenstone and Bennett, 1980; Anderson, 1994). For example, Anderson (1994), analyzing species of the family Theridiidae with different life habits, only found differences attributable to food restriction. However, Shillington (2005) found higher resting metabolic rates in the more active males than females of *Aphonopelma anax*, suggesting that sexual differences in the habits of this spider could explain the metabolic differences. Recently Kawamoto et al. (2011) contradicted the idea that spiders can be understood as land arthropods in energetic terms (Lighton et al., 2001), showing allometric differences in resting metabolic rates between ecribellate and cribellate orb weaver spiders, probably due to behavioral and activity differences associated with web building.

Carrel and Heathcote (1976), based on the low heart rate of primitive hunter and weaver spiders (Loxoscelidae (Sicariidae) and Scytodidae), proposed the hypothesis that these groups would have low metabolic rates, suggesting that this would be an energy-conserving adaptation of spiders that invest little effort in prey capture, and consequently feed only occasionally. However, Greenstone and Bennett (1980) did not find metabolic differences other than those due to body mass between spiders of the genus *Loxosceles* and other spiders. While Carrel and Heathcote (1976) proposed that the nearly constant ratio of 2.5 between heart rate and metabolism suggested that Sicariidae and Scytodidae would have a low resting metabolic rate, Greenstone and Bennett (1980) rejected this idea and suggested that heart rate is an unreliable predictor of metabolic rate.

**Figure 1:**
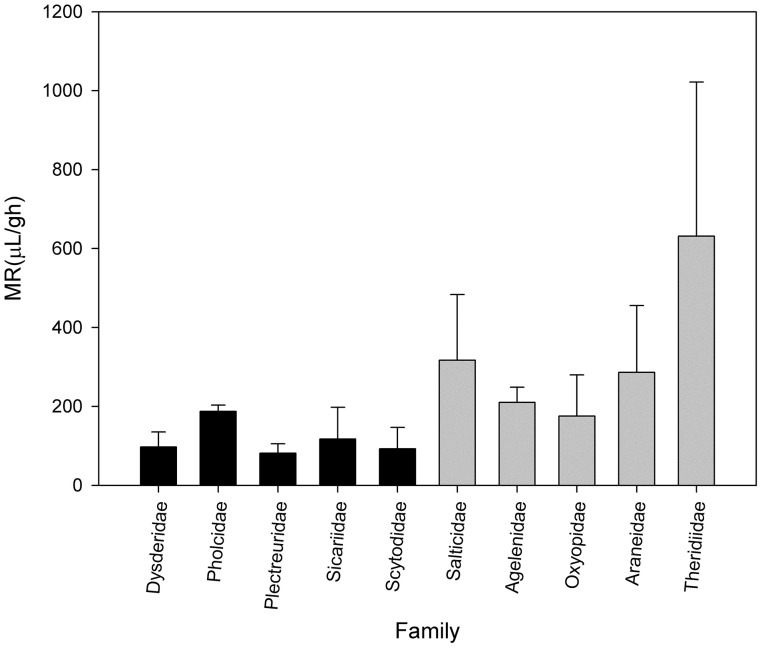
**Metabolic rate of some families of spiders of comparable body mass**. Black bars represents primitive hunters and weavers haplogyne spiders. Gray bars represents modern spiders such as Salticidae and orb-weaving spiders (Data from Greenstone and Bennett, 1980; Canals et al., 2015).

Canals et al. (2015) analyzed the metabolic rate of the primitive hunters and weavers *Loxosceles laeta* (Sicariidae) and *Scytodes globula* (Scytodidae) and their relationship to body mass, comparing with metabolic data of other spiders. They found a low metabolic rate in these species and other primitive hunters and weavers such as spiders of the families Dysderidae and Plectreuridae. The metabolic rate of this group was lower than in other non-primitive spiders such as orb weavers, rejecting the proposition of a general relationship for all land arthropods. This agree with the Carrel and Heathcote hypothesis, suggesting that metabolic rates not only are affected by sex, reproductive and developmental status but also by ecology and life style, recognizing at least in the araneomorph spiders a group with low metabolism composed of the primitive hunters and weavers and another composed of the web building spiders (Figure 1).

## Temperature

Climate, in particular environmental temperature, affects virtually every aspect of the life of arthropods; it was called “the weather school” in a classic work of insect ecology (Andrewartha and Birch, 1954). The weather influences many aspects of life history and habitat selection, mating and development (Canals, 1998; Angilletta et al., 2002).

Thermal biology has been poorly studied in spiders, limited to the study of the thermal limits of few species and their relationship with the habitat (Humphreys, 1987; Schmalhofer, 1999; Hanna and Cobb, 2007). Environmental temperature is particularly relevant in spiders, since the thermal tolerance limits may help them choose suitable mating and foraging sites, particularly in species in which the female remains in the shelter with her egg sac (Hanna and Cobb, 2007).

Veloso et al. (2012) studied the thermal biology of *P. parvula*, a small mygalomorph spider inhabiting the high Andes. They found that this species has a preferred temperature of about 31°C, similar to that reported for *Aphonopelma* sp. between 27 and 35°C (Seymour and Vinegar, 1973) and found no differences between juveniles and adults, which contrasts with previous results reported for araneomorph spiders of the family Lycosidae (Sevacherian and Lowrie, 1972; Humphreys, 1975, 1977, 1978; De Vito and Formanowics, 2003). For example, Sevacherian and Lowrie (1972) found that juveniles of two species of the genus *Pardosa* had optimum temperatures lower than those of adults of the same species, and De Vito and Formanowics (2003) found that juvenile individuals of *Pirata sedentarius* species exposed to heat stress survive better than adults. Females of *P. parvula* with egg sacs had preferred temperatures 3°C lower than non-breeding females. In this respect other authors have found higher preferred temperatures in reproductive females (Norgaard, 1951; Vlijm et al., 1963; Frick et al., 2007) compared to non-reproductive individuals. The study of Veloso et al. (2012) also demonstrated that *P. parvula* thermoregulates behaviorally, changing its temperature at a rate slower than the temperature of the stones, air and substrate, but always with body temperatures better explained by the temperature of the substrate where the spider is, similar to that reported for *Aphonopelma* sp. (Seymour and Vinegar, 1973).

Increments in preferred temperatures during the day have been reported in *G. rosea* and *P. parvula*, which is probably associated with their crepuscular and nocturnal activity. The former is large (>10 g) and inhabits arid and semi-arid areas near plant communities composed of shrubs and small trees, while the second lives in high altitude habitats at about 2000 m in rocky environments. The smaller spider presented a negative relationship between the preferred temperature and body size, while *G. rosea* did not show this negative association. In addition, the preferred temperature of *P. parvula* was higher than that of *G. rosea*. These results suggest greater susceptibility to the environmental temperature in small spiders. Acclimating these spiders to cold (15°C) and heat (25°C), an increment of 2–3°C has been found in preferred temperatures, but only in *P. parvula*. Thus, *G. rosea*, and *P. parvula* represent two different strategies, one with small body size in fluctuating environments and high altitude with physiological plasticity and the other large with high thermal inertia adapted to the more stable environments of the lowlands (Alfaro et al., 2013a).

**Figure 2:**
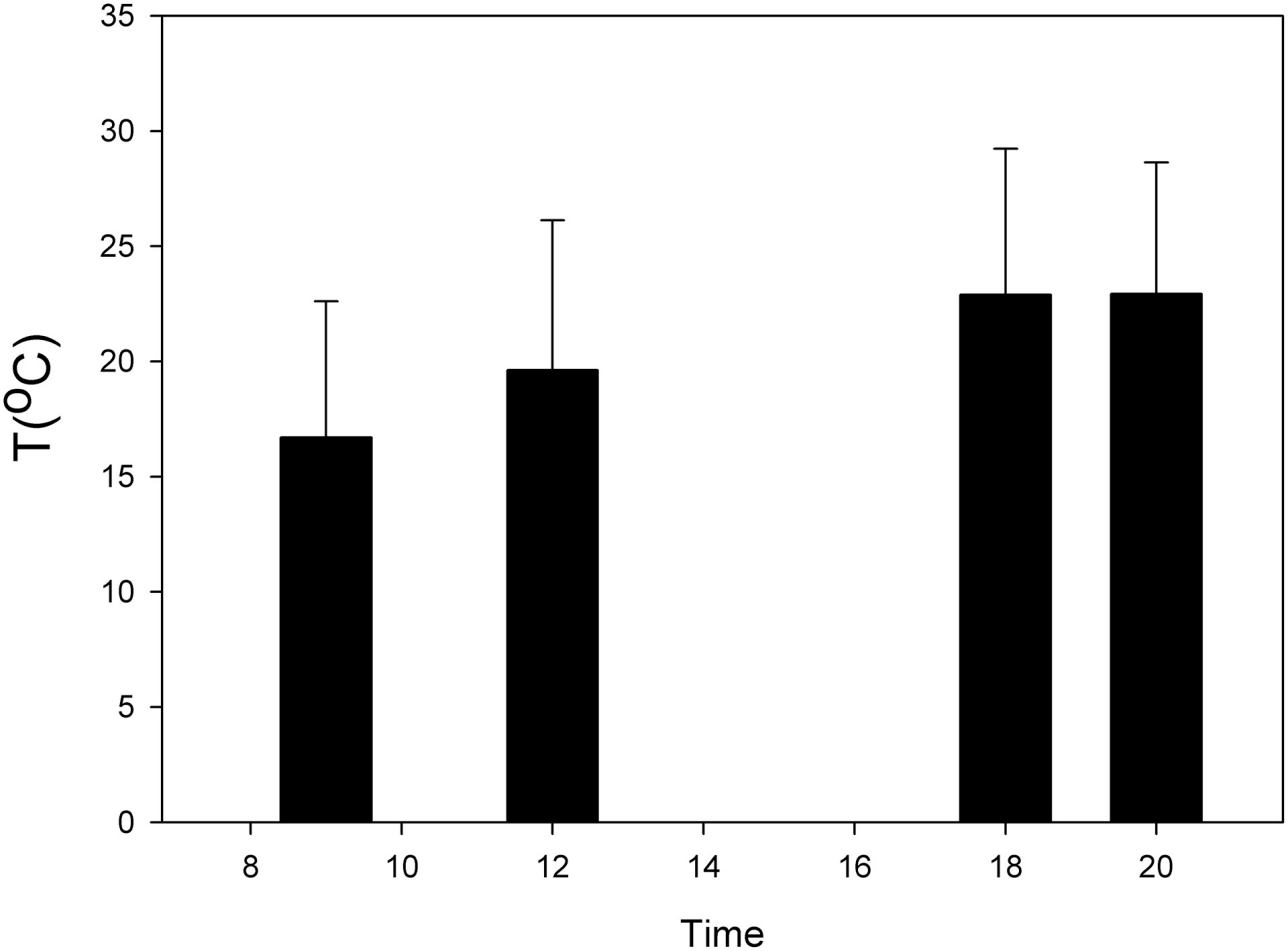
**Daily variation of temperature of the nocturnal spider *Loxosceles laeta* (modified from Alfaro et al., 2013b)**.

In the Chilean recluse spider *Loxosceles laeta* and the spitting spider *Scytodes globula* preferred temperatures (Tp) higher at twilight have been reported (Alfaro et al., 2013b) (Figure 2). The thermal niche breadth (Ba) of *S. globula* and *L. laeta* is similar (Ba = 0.62 and 0.61, respectively) and they have a wide thermal niche overlap (Alfaro et al., 2013b; Canals et al., 2013). Minimum critical temperatures in the two species were less than −3°C and maximum critical temperatures were over 45°C, which indicates a wide zone of tolerance in these spiders (Alfaro et al., 2013b). These results are similar to that reported in other *Loxosceles* species (Fisher and Vasconcellos-Neto, 2003; Cramer and Maywright, 2008).

The araneomorphae *Dysdera crocata* that inhabits central Chile sharing its habitat with the common woodlouse *Porcellio laevis*, its usual prey. These two species that share a habitat in the field, one a predator and the other its prey, have similar preferred temperatures: 9.12 ± 5.12°C and 9.4 ± 1.1°C. In contrast with *L. laeta* and *S. globula, D. crocata* did not present hourly variations in preferred temperature throughout the day. Regarding thermal preferences, *D. crocata* had a more narrow range of thermal microenvironment preference than these species. The election of low temperatures and a relatively narrow range may be explained by phenotypic plasticity as an adaptation to the particular environmental conditions present in Chile (Sepulveda et al., 2014). Species from different environments typically also have different thermal preferences (Schmalhofer, 1999) and these may vary seasonally (Schmalhofer, 1999), with the breeding season (Hanna and Cobb, 2007; Veloso et al., 2012) or during the day, as in other ectothermic animals (Canals et al., 1997b; Alfaro et al., 2013b).

## Water balance and desiccation

The high surface/volume of terrestrial arthropods makes them susceptible to rapid heat loss and gain and high water loss, especially under conditions of high temperature and low relative humidity, creating conflicts for water conservation. Strictly related to the thermal environment, strategies for water loss in spiders may be part of a set of physiological adaptations to tolerate unpredictable environments (Canals et al., 2011). Desiccation is a significant stress to terrestrial arthropods and numerous mechanisms have been proposed to increase desiccation tolerance, including: (i) reduction in the rate of water loss, (ii) increased water content, and (iii) increase in desiccation tolerance (Gibbs et al., 1997; Gibbs, 2002; Bazinet et al., 2010). For example, it has been proposed that in insects the loss of respiratory water can be reduced by controlling breathing patterns (Chown, 2002; White et al., 2007) and loss of cuticle water may be controlled by variations in permeability or in epicuticular carbon hydrates (Gibbs, 2002). Water can be increased by increasing the volume of hemolymph (Hadley, 1994) or by accumulation of glycogen (Gibbs, 2002). The mechanisms underlying tolerance to loss of water are not well understood, although a role of trehalose and heat shock proteins has been proposed in cell protection of organism that survive losing large amounts of water (Watanabe, 2006; Benoit et al., 2010). It has also been proposed that photoperiodic diapause can increase desiccation resistance in insects. For example, *Aedes albopictus* eggs in diapause have 33% of the carbohydrate loss and half the water loss of eggs which are not in diapause (Urbanski et al., 2010). Some studies have examined the relationship between loss of water and the environment in which the species live (Vollmer and Mac Mahon, 1974; Edney, 1977; Hadley et al., 1981; Hadley and Quinlan, 1989). For example Hadley et al. (1981) reported that spiders of xeric environments have lower rates of evaporation than spiders that live in caves, and Hadley and Quinlan (1989) suggested that the low rate of evaporation in the black widow (*Latrodectus heperus*) allowed it to invade desert habitats in North America.

Like temperature, desiccation tolerance has a strict connection with the microhabitat of spiders (Vollmer and Mac Mahon, 1974; De Vito and Formanowics, 2003; De Vito et al., 2004). For example De Vito et al. (2004) compared the tolerance to heat stress and desiccation of three species of wolf spiders (Lycosidae) *Pirata sedentarius, Pardosa lapidicina*, and *Pardosa fuscula* and correlated it with the microhabitat of each species, finding that *P. sedentarius* has lower tolerance to desiccation and is now more restricted to the proximity of water than the two species of *Pardosa*.

In mygalomorph spiders, breathing gases involves moving air along surface exchange and combining with the circulating respiratory pigment, hemocyanin (Anderson and Prestwich, 1982; Paul et al., 1987). Gas exchange occurs in the book lungs through a thin cuticle hypodermic gas barrier which separates the atrium from the hemolymph (Canals et al., 2008). Spiracles are highly complex openings of the respiratory system to the environment that can be opened or closed to allow a variable amount of gas exchange. It is proposed that the detailed control helps prevent water loss. Spiracles are opened more frequently and more extensively at high temperatures and with increased activity of the body, according to the increased need of oxygen (Schmidt-Nielsen, 1997). The ventilation of the respiratory system, especially the function of the spiracles, is influenced by the presence of carbon dioxide (Schmidt-Nielsen, 1997) being the main stimulus for opening (Davies and Edney, 1952; Figueroa et al., 2010). The required concentration of carbon dioxide to open the spiracles is quite low; in the cockroach, for example, 1% carbon dioxide in air shows a perceptible effect; 2% makes spiracles remain open and 3% makes them stay wide open (Schmidt-Nielsen, 1997). Lighton et al. (2001) proposed that in resting insects the degree of spiracular opening is modulated by the partial pressure of oxygen in addition to carbon dioxide. Spiracles of the lungs in book spiders are almost closed in the animal at rest (Davies and Edney, 1952).

The lungs of mygalomorph spiders have been reported as highly refined. For example, in *G. rosea* the harmonic mean of the air-barrier lymph is 0.14 ± 0.03 μm, with high respiratory surface and a large lung volume, which determines a high oxygen diffusion capacity. Thus, a difference of oxygen partial pressure of only 0.12–0.16 kPa is sufficient to sustain their energy requirements, which is very impressive compared to about 7.5 kPa that mammals require (Canals et al., 2008) (Table 2).

**Table 2:** **Respiratory refinements of the spiders *Grammostola rosea* (Theraphosidae) (Canals et al., 2008), *Salticus scenicus* (Salticidae) (Strazny and Perry, 1984) and *Tegenaria* sp. (Agelenidae) (Schmitz and Perry, 2001)**.

	**τ_h_(μm)**	**RS_d_ (mm^−1^)**	**DtO_2_ (cm^3^/ghkPa)**	**ΔPO_2_ (kPa)**
*Grammostola rosea*	0.14 ± 0.03	122.99 ± 35.84	0.233-0.552	0.160-0.119
*Salticus scenicus*	0.10 - 0.18	210 - 250	0.700 - 0.984	0.220 - 0.260
*Tegenaria* sp.	0.4	350 - 390	0.258 - 0.552	0.380 - 0.810

*Where τ_h_ is the harmonic mean thickness of the air-blodd barrier, RS_d_ the respiratory surface density, DtO_2_ is the tissue oxygen diffusion capacity and ΔPO_2_ is the difference of partial oxygen pressures needed to overcome the resting metabolic rate*.

**Figure 3:**
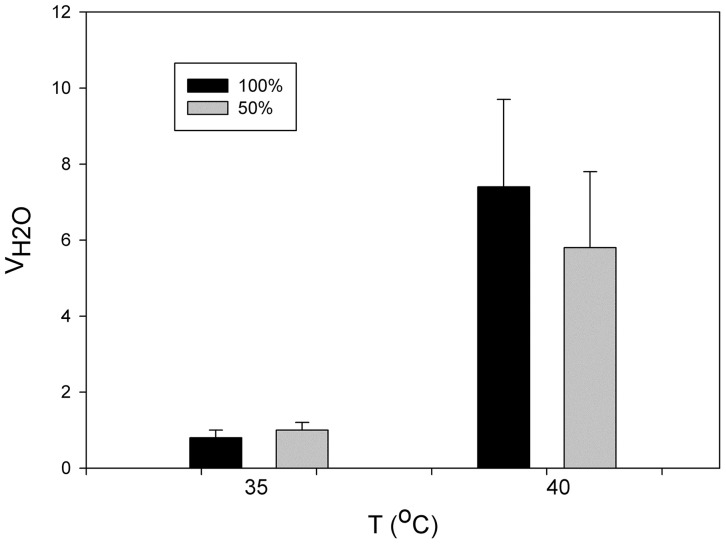
**Variation in the mass specific evaporative water loss (V_H2O_) as function of temperature in the mygalomorphae spider *Paraphysa parvula* with 50% (gray bars) and 100% (black) of spiracles open (modified from Figueroa et al., 2010)**.

The rate of evaporative water loss of *P. parvula* increases dramatically, about 10-fold, at temperatures near 40°C, a temperature that must be close to its thermal limits. In this same spider the participation of book lungs in evaporative water loss was estimated to be 60% (Figueroa et al., 2010) (Figure 3). In other invertebrates, the effect of open spiracles in water loss has been reported as considerable. For example, in the earthworm, which spends its entire life in the dry soil environment, controlling the spiracles is very important. If the spiracles are open water loss immediately increases by several times and can cause death (Schmidt-Nielsen, 1997).

The metabolic rate of *P. parvula* was not affected by water restriction. In addition, there were seasonal changes in evaporative water loss, which was higher at the time of lower temperatures, and there was lower water loss in reproductive females carrying their egg sac. These findings constitute adaptations of this spider to high Mediterranean environments, wildly fluctuating, completely covered with snow in winter and extremely hot in summer (Canals et al., 2011). Also, in the araneomorph spiders *L. laeta* and *S. globula* lower rates of water loss in summer than in winter has been reported, which has been interpreted as an adaptation to xeric environments probably mediated by changes in the cuticle permeability (Canals et al., 2013).

## Concluding remarks

The ecophysiology of spiders is deeply influenced by three factors: the ectothermy, the body size, and the presence of the cuticle. Ectothermy on the one hand makes the metabolism of spiders dependent external environment determining the need for behavioral thermoregulation, but also minimizes heat loss by reducing the thermal differential between body and the environment. The small body size on the one hand involves a susceptibility of these organisms by the large area (relative to body size) of the surface for water and heat exchange with the external environment, but ensures lower energy expenditure and lower cost of transportation (Grossi and Canals, 2015). The cuticle on the one hand limits the body size but then prevents excessive water loss.

Many aspects of spider physiology suggest a strategy of minimum energy expenditure, such as external digestion consequences on specific dynamic action (Nespolo et al., 2011), the refinement of the book lungs (Canals et al., 2008) and their low metabolism (Canals et al., 2015).

The case of Chilean spiders shows how the ecophysiology in spiders is associated with their design and body size and how it is affected by fluctuating Mediterranean environments, suggesting that the adaptive process can be seen as a route of optimization in the use of energy to cope with environmental restrictions imposed by the interaction with the terrestrial environment and lifestyle.

### Conflict of interest statement

The authors declare that the research was conducted in the absence of any commercial or financial relationships that could be construed as a potential conflict of interest.
